# Meta-analysis of the growth rates of abdominal aortic aneurysm in the Chinese population

**DOI:** 10.1186/s12872-019-1160-x

**Published:** 2019-08-22

**Authors:** Tingting Huang, Shuai Liu, Jianhua Huang, Baohui Xu, Yongping Bai, Wei Wang

**Affiliations:** 1 Department of Vascular Surgery and Department of Cardiology, Xiangya Hospital, Central South University, Changsha, 410000 Hunan China; 2 Department of Vascular Surgery, Xiangya Hospital, Central South University, Changsha, 410000 Hunan China; 3 Department of Vascular Surgery, Stanford University School of Medicine, Stanford, California 94305 USA; 4 Department of Cardiology, Xiangya Hospital, Central 27 South University, Changsha, Hunan China

**Keywords:** Abdominal aortic aneurysm, Growth rate, Meta-analysis

## Abstract

**Background:**

Several studies on the growth rates of abdominal aortic aneurysm (AAA) in Chinese population have been conducted; however, this issue remains unclear. The aim of this study is to systematically review published data of the AAA growth rates among people in China.

**Methods:**

We conducted a comprehensive search of multiple databases to identify all studies of AAA growth in the Chinese population from inception until June 2017. AAA growth rates were combined to yield the growth rates at specified aneurysm diameter ranges, with using a random-effects model or fixed-effects model according to heterogeneity.

**Results:**

A total of 8257 studies were initially identified and only 4 studies were eventually included. A random-effects analysis showed that the growth rates of AAA in Chinses population is ranging from 0.18 cm/year to 0.75 cm/year. The pooled mean growth rates among individuals with aneurysm measuring 3.0–3.9 cm, 4.0–5.9 cm and ≧ 6.0 cm in diameter were 0.21 cm/year (95% CI: 0.19 cm/year to 0.23 cm/year), 0.38 cm/year (95% CI: 0.33 cm/year to 0.43 cm/year), and 0.71 cm/year (95% CI: 0.64 cm/year to 0.77 cm/year) respectively. Further analysis found that the pooled mean growth rates for individuals with small AAA (diameters measuring 3.0–4.9 cm) was 0.28 cm/year (95% CI: − 0.06 cm/year to 0.61 cm/year)`and for individuals with large AAA (diameters ≥5.0 cm) was 0.75 cm/year (95% CI: 0.20 cm/year to 1.3 cm/year). Finally, meta-regression showed a strong trend of linear relationship between AAA growth rate and aneurysm diameter.

**Conclusions:**

The growth rates of AAA in the Chinese population increase with AAA enlargement and appear to range from 0.18 cm/year in the smallest AAAs to 0.75 cm/year when the diameter exceeds 6 cm. However, based on current studies, it is difficult to estimate the accurate average AAA growth rate in Chinese patients. More large-scale, high-quality studies are required to achieve that. Overall, AAA growth rate increase with increased aneurysm diameter.

**Electronic supplementary material:**

The online version of this article (10.1186/s12872-019-1160-x) contains supplementary material, which is available to authorized users.

## Background

Abdominal aortic aneurysm (AAA) refers to an abdominal aortic dilation, with an aortic diameter greater than 3.0 cm or 1.5 times the expected normal diameter [1–3]. AAA is caused by degeneration of the elastic tissue and depletion of vascular smooth muscle in the arterial media [1–5]. The prevalence rates of AAA are negligible before the age of 50–60 years and after that the prevalence rates vary between 1.3% and over 5% [6]. The risk factors for AAA includes male sex, smoking, older age, ethnicity, atherosclerosis, hypertension and family history [1, 6–10]. The condition is usually asymptomatic before rupture, but the mortality rate reaches 90% when rupture occurs [11, 12]. Currently, although many scholars including our team have done a lot of work and trial in the field of pathogenesis of AAA and drug therapy for AAA [13–17], surgical treatment (including open surgical treatment and endovascular repair) is the only effective way to treat AAA [18]. The threshold for performing elective surgery is aneurysm diameter of 5.5 cm [6, 19]; when the diameter is less than 5.5 cm, the survival rate of patients who are continually monitored is comparable to that of patients who undergo elective surgery [9, 20, 21].

The management plan of AAA has been established on the basis of data for estimated growth rate, estimated rupture rate, and estimated risk of a surgical procedure, nearly all of which have been acquired from European and American populations [7, 9, 20–22]. Previous studies have revealed that white populations, especially white males, have a greater prevalence and incidence of AAA compared with black populations [23, 24]. One study reported that race was not an independent predictor of mortality after surgery for AAA, and the difference in observed mortality rates among white and black patients was caused by preoperative risk factors [25]. However, another study reported that Hispanic ethnicity was independently associated with increased mortality after repair of thoracoabdominal aneurysm [26]. Moreover, morphological features of AAA are significantly different among white and Asian patients [27]. Taken together, we can safely speculate that differences exist among patients of different races with AAA. Therefore, it is inappropriate to manage Chinese patients with AAA according to guidelines developed on the basis of data acquired from European and American patients, especially for the surveillance intervals management of Chinese patients with AAA [28, 29]. To address this issue, our meta-analysis aims to assess the growth rate of AAA in the Chinese population, to provide clues for clinical practice.

## Methods

### Search strategies and selection criteria

This systematic review followed the quality reporting guideline set by the Preferred Reporting Items for Systematic Reviews and Meta-Analyses (PRISMA) group [30]. English databases, including MEDLINE, EMBASE, the Cochrane Library, and Chinese databases, including CBM, CNKI, VIP and Wanfang were searched from inception until June 2017. We referred to a study by Thompson for search strategies used in English databases [31]. When searching Chinese databases, we used the following terms: abdominal aortic aneurysm, growth, screen, surveillance, and follow-up. Reference lists of relevant studies were manually searched. The inclusion criteria were as follows: studies among Chinese participants with infrarenal AAA, assessed by either ultrasound (US) or computed tomography (CT) scan on at least two occasions at least 6 months apart. Review papers, case reports, studies in which patient data were duplicated, non-human studies, studies among patients previously treated by AAA surgery, and studies including patients with Marfan syndrome were excluded.

### Study selection and data extraction

Studies were identified using the abovementioned search strategies. Data extraction was performed by two independent reviewers. Where there was any disagreement, a third reviewer was consulted. The following information/data were extracted from studies that met the inclusion criteria: year of publication, study design, method of follow-up (US, CT), frequency of follow-up, participant information (number of participants, age, sex, other cardiovascular risk factors), length of follow-up, inclusion criteria, recruitment period, trial using a particular drug, outcomes (how was aortic diameter measured, whether growth was reported by size band, number of non-AAA deaths, number of AAA repairs, who carried out aortic measurements), and analysis.

### Quality assessment

Quality assessment was performed by two reviewers independently and a third reviewer was consulted in cases of any disagreement. There is no widely recognized quality assessment tools for single-group observational studies, and we had to relax the eligibility criteria for studies because related studies are very limited, so we only performed a basic quality assessment. The criteria for quality control included: type of study, standardization of imaging, description of outcomes, and reporting methods (graphic, descriptive, tables, statistical uncertainty). Quality assessment of the included studies is shown in Table 1.

**Table 1:** Quality assessment of studies included in the systematic review

Study	Study type (P, R, PR)	Study type (RCT/obs)	Standardisation of imaging	Variability of diameter given	Patient selection criteria
Li, J., 2008	R	Obs	Y	N	All patients diagnosed as AAA during 1975 and 2007 in the Department of Geriatric Medicine of Beijing Hospital with imaging proof (US, CT or MRI)
Fan, L.H. 1999	Unclear	Obs	N	N	Not described
Wu, Q., 2009	R	Obs	N	N	All patients aged above 80 and diagnosed as AAA during January, 1997 and Juanuary, 2008 in People’s Liberation Army General Hospital
Zhang, L.F., 2006	P	Obs	Y	N	All patients whose medical history and imaging data are complete and aneurysm had been regularly monitored after being diagnosed as AAA in Air Force General Hospital.
Zhao, B. 2008	P	Obs	N	N	Randomly selected from outpatients and inpatients diagnosed with AAA during US screening, and with no syptom or sign.
Song, H.G., 2013	R	Obs	N	N	Not described.

Abbreviations: *N* no, *U* unclear, *Y* yes, *Obs* observational study, *P* prospective, *PR* prospective and retrospective, *R* retrospective, *RCT* randomized controlled trial

### Data synthesis and analysis

Reported overall mean growth rates (mm/year) and its standard deviation (SD) were extracted from each study. If growth was reported by size bands, growth rates in size bands were also extracted. If growth rates were reported in size bands only without reporting of the overall growth rate, the size band estimates were pooled, using formulas M= (*N*_1_*M*_1_ + *N*_2_*M*_2_)/(*N*_1_ + *N*_2_) and $\mathrm{SD}=\sqrt{\frac{\left({N}_1-1\right){SD_1}^2+\left({N}_2-1\right){SD_2}^2+\frac{N_1{N}_2}{N_1+{N}_2}\left({M_1}^2+{M_2}^2-2{M}_1{M}_2\right)}{N_1+{N}_2-1}},$ to obtain an additional overall growth rate.

Heterogeneity among included studies was assessed by determining the I^2^ statistic. If I^2^ < 50%, a fixed-effects model was used for meta-analysis; otherwise, a random-effects model was used. Publication bias analysis was only to be performed if there were more than 10 included studies.

Sensitivity analysis of the influence of each study on the pooled estimate for aneurysm growth rate was performed by excluding individual studies. Subgroup meta-analyses for growth rates reported by size range were also conducted. Meta-regression was conducted to further investigate the source of heterogeneity and the relationship between aneurysm diameter and AAA growth rate. Statistical analysis was performed using Stata software, version 12 (StataCorp LLC. College Station, TX, USA).

### Ethical approval statement

All analyses were based on previous published studies; therefore, no ethics approval or patient consent was required.

## Results

### Literature search

**Fig. 1:**
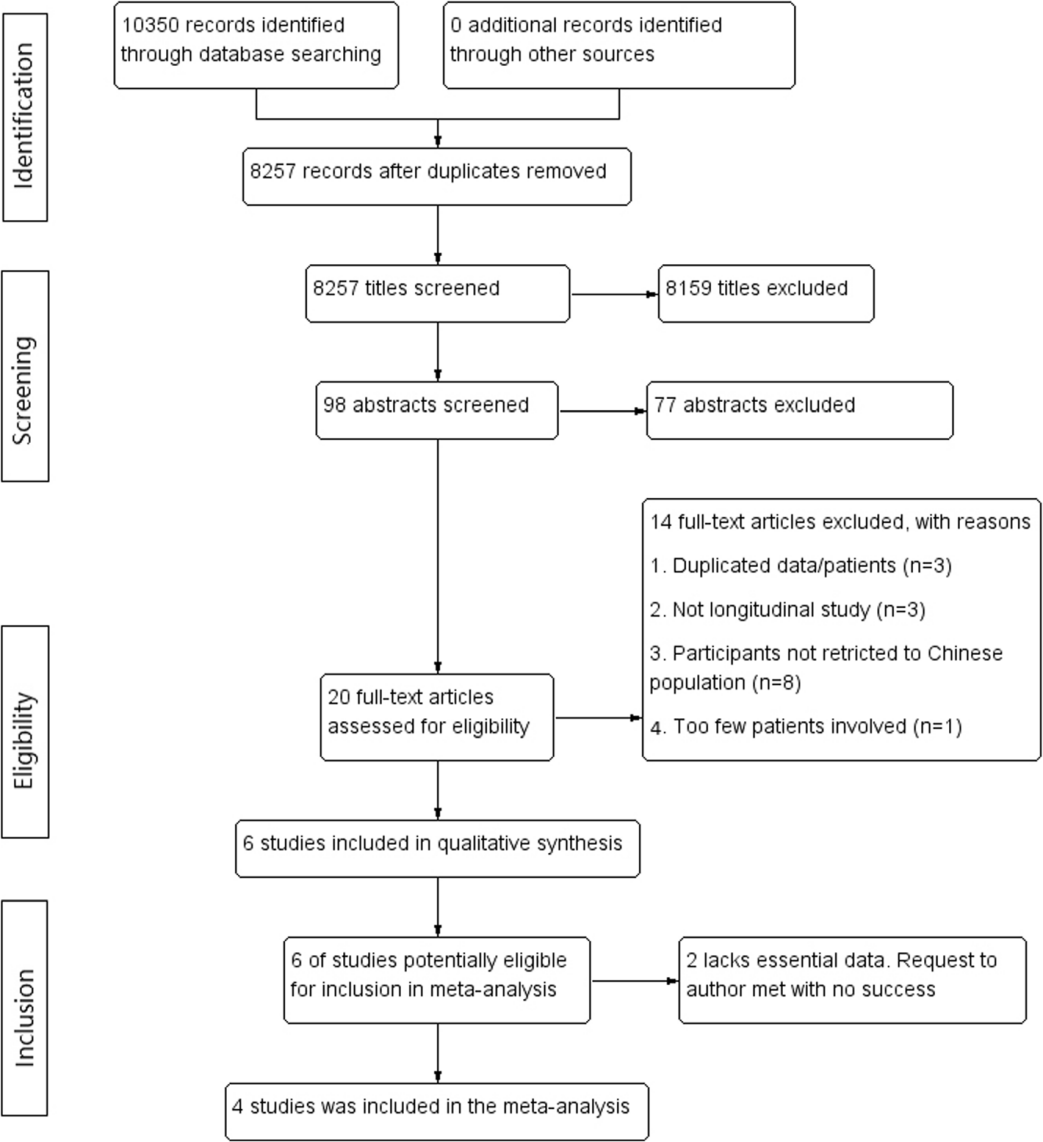
Study flow diagram. A total of 8257 studies were initially identified and only 4 studies were included for meta-analysis

A study flow diagram is shown in Fig. 1. A total of 8257 studies were initially identified; 8159 were excluded after title screening and 77 were excluded after abstract screening. Twenty full-text articles were assessed, and 14 were excluded (details are shown in Additional file 1: Table S1). Two of the remaining six studies were excluded because one lacked SD or related information from which SD could be calculated; the other study reported the AAA growth range but not growth rate [32, 33]. Requests made to the author for additional information went unanswered.

### Study characteristics

The characteristics of the four included studies are summarized in Table 2. The publication dates of the four studies ranged from 2006 to 2013. All were are observational studies [34–37] and three were retrospective studies [35–37]. The study size ranged from 37 to 78 patients, with 3.8 to 43.8% female participants. All four studies used US to monitor aortic diameter, measuring the maximum external cross-sectional direction in any plane. The length of follow-up ranged from 0.5 to 25 years. Three studies included patients with aortic diameters greater than 3.0 cm [34, 36, 37], and one study reported aortic diameters greater than 2.1 cm [35]. Aneurysm growth rate was the primary outcome measure in the four studies; however, the methods used to estimate growth rates were not reported. All four studies reported aneurysm growth rates by size bands; only one study reported growth rates by age bands [35].

**Table 2:** Characteristics of included studies

First author, year	Study type	Number of patients (women)	US/CT	Aortic diameter measured	Frequency of follow-up	Cardiovascular risks	Length of follow-up (enrolment dates)	Method of growth rate estimating and reporting	Baseline AAA/cm	Growth rates cm/year
Li, J., 2008	Obs R	78 (3)	US	Maximal external cross-sectional in any plane	Once a year	Hypertension (*n* = 57) DM (*n* = 18) CHD (*n* = 62) MI (*n* = 32) Hypercholesterolemia (*n* = 44) Smoking (*n* = 52)	1~25 years, mean 7.9 ± 5.5 years (1975–2007)	Not reported; x ± sd	Overall: 2.1–5.7 (*n* = 64), [Growth rate only calculated for 64 patients	0.18 ± 0.23
									2.1–2.9 (*n* = 27)	0.13 ± 0.16
									3.0–4.9 (*n* = 30)	0.09 ± 0.07
									≧5.0 (*n* = 14)	0.46 ± 0.33
Wu, Q., 2009	Obs R	66 (4)	US	Maximal external cross-sectional in any plane	unclear	Hypertension (*n* = 66) DM (*n* = 28) CHD (*n* = 62) Hyperglycemia (*n* = 61) Peripheral atherosclerosis (*n* = 63) CI (*n* = 30) Smoking (*n* = 62)	11 years maximum (1997–2008)	Not reported; x ± sd	Overall:3.0–9.2 (*n* = 66)	0.75 ± 0.34
									3.0~4.9 (*n* = 31)	0.45 ± 0.24
									≧5.0 cm (*n* = 35)	1.02 ± 0.10
Zhang, L.F., 2006	Obs P	37 (4)	US	Maximal external cross-sectional in any plane	unclear	Hypertension (*n* = 21) CHD (*n* = 17) DM (*n* = 7) CI (*n* = 11) CRF (*n* = 3) PHD (*n* = 3) Other (*n* = 9)	0.5~11 years, mean 6.1 (unclear)	Not reported; x ± sd	Overall:3.0–8.9 (*n* = 37)	X = 0.38 ± 0.26
									3.0~3.9 (*n* = 14)	0.23 ± 0.11
									4.0~5.9 (*n* = 15)	0.37 ± 0.24
									6.0~8.9 (*n* = 8)	0.67 ± 0.29
Song, H.G., 2013	Obs R	57 (25)	US	Maximal external cross-sectional in any plane	Unclear	Unclear	1~8 years, mean 4.1 ± 1.6 years (unclear)	Not reported;x ± sd	Overall: unclear (*n* = 57)	0.40 ± 0.22
									3.0–3.9 (n = 21)	0.21 ± 0.05
									4.00~5.9 (*n* = 22)	0.38 ± 0.13
									> 6.0 (n = 14)	0.71 ± 0.14

Abbreviations: *Obs* observational study, *P* prospective study, *R* retrospective study, *US* ultrasonography, *CT* computed tomography

### Outcome analysis

**Fig. 2:**
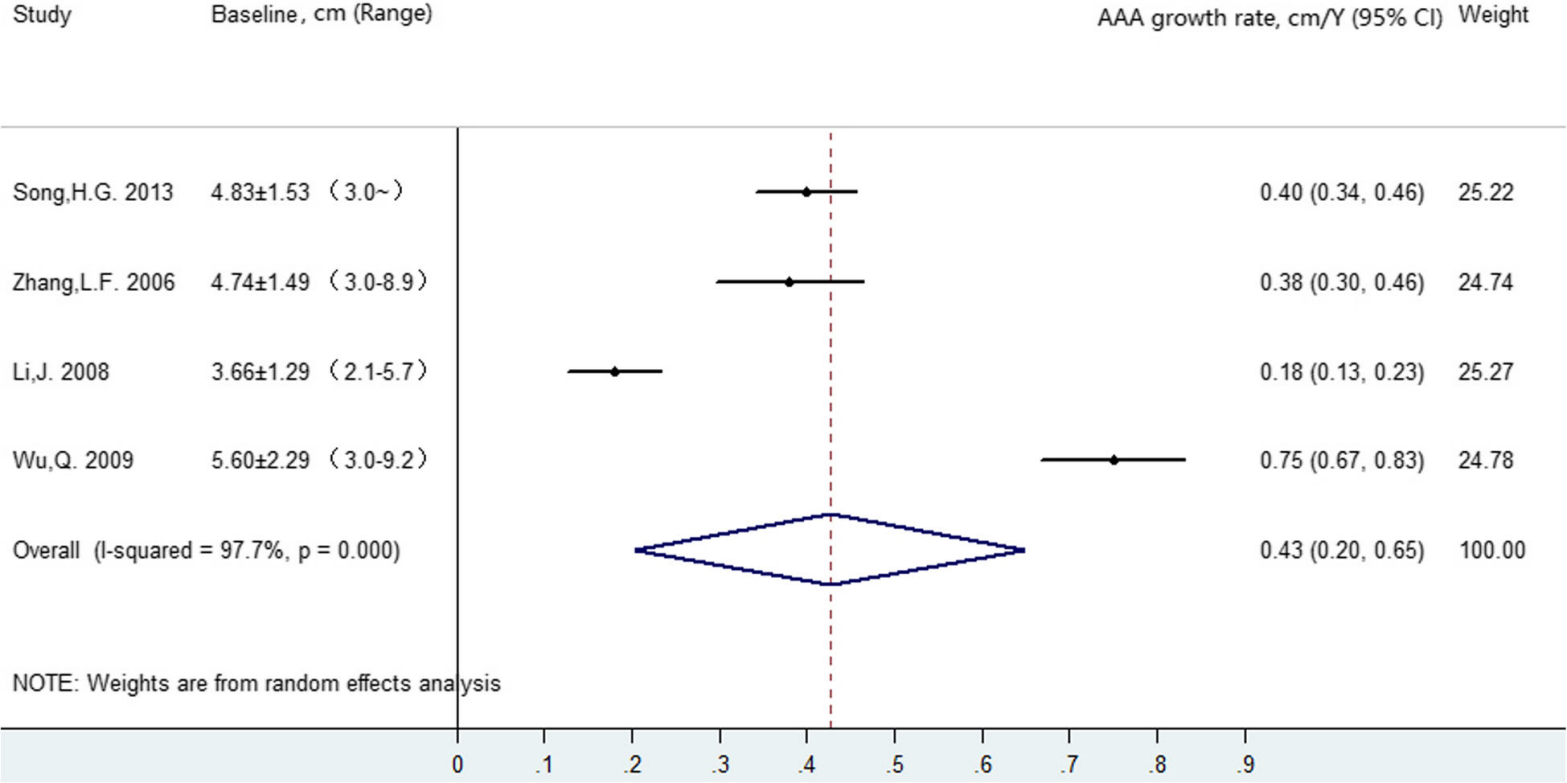
Overall AAA growth rate estimates

A forest plot summarizing overall average growth rate estimates is shown in Fig. 2. A random-effects analysis was applied owing to the substantial heterogeneity between studies (I^2^ =97.7, 95% confidence interval (CI): 96 to 99%), with growth rates ranging from 0.18 cm/year to 0.75 cm/year. Because one study included aortic diameters greater than 2.1 cm whereas the remaining three studies reported diameters greater than 3.0 cm, meta-analysis was performed after excluding data acquired from patients with aneurysm diameter less than 3.0 cm. The heterogeneity (I^2^ =96.7, 95% CI: 94 to 98%) remained substantial (figure not shown), and the random-effects meta-analysis estimate of the average AAA growth rate was 0.43 cm/year (95% CI: 0.23 cm/year to 0.64 cm/year).

We performed a sensitivity analysis of the influence of each study on the pooled estimate for aneurysm growth rate (figure not shown). The pooled AAA growth rates (0.32 cm/year to 0.51 cm/year) and heterogeneity (I^2^ ranging from 94.2 to 98.5%) did not change markedly with the exclusion of individual studies, indicating that the meta-analysis was relatively reliable.

**Fig. 3:**
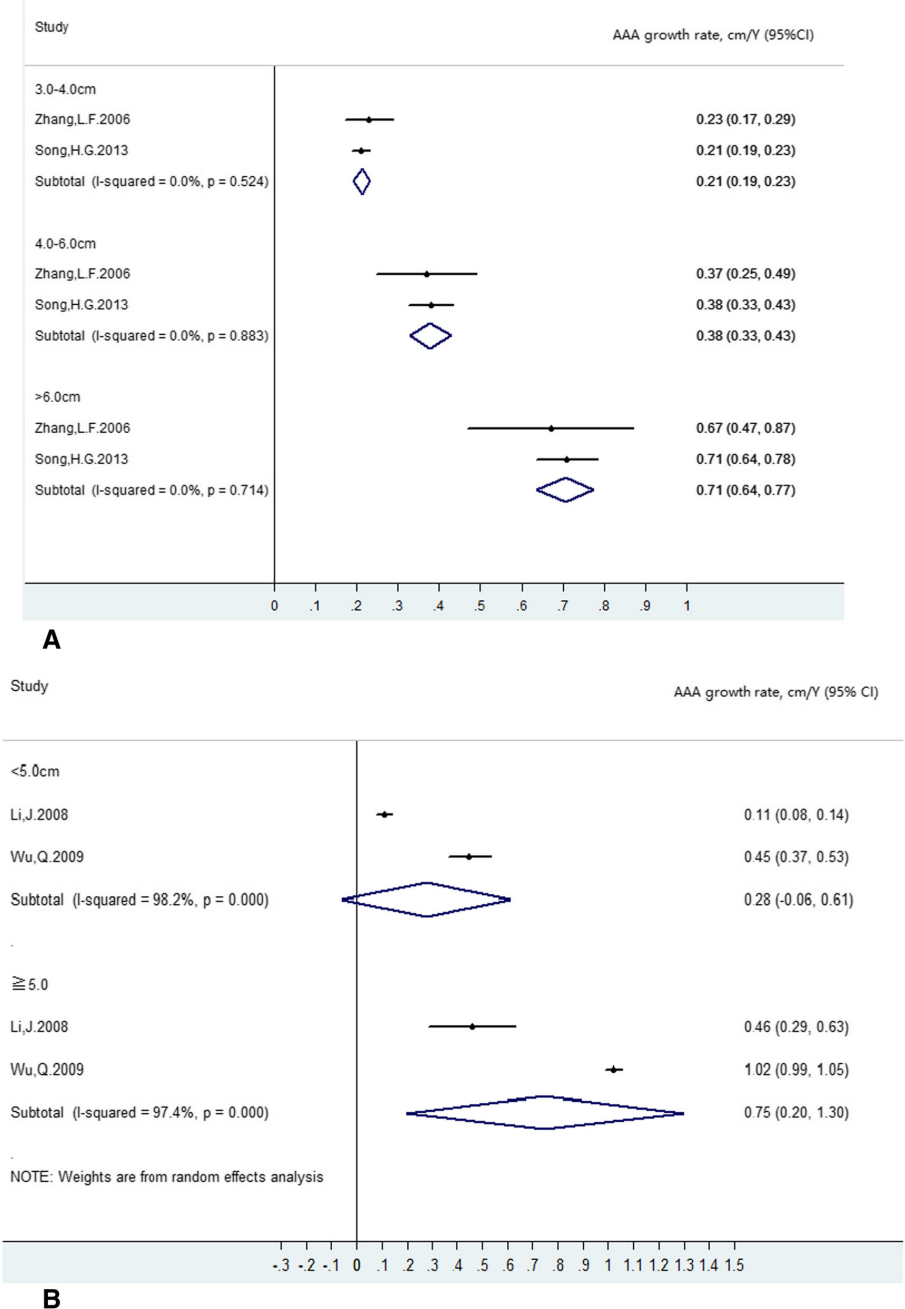
**a** Baseline diameters: 3.0–3.9 cm, 4.0–5.9 cm and ≥6.0 cm. Fixed-effects model was applied. **b** Baseline diameters: 3.0–4.9 cm and ≥5.0 cm. Ransom-effects model was applied

In two studies [34, 37], AAA diameters at baseline were divided into 3.0–3.9 cm, 4.0–5.9 cm, and ≥ 6.0 cm. Estimates of AAA growth rate within these ranges are summarized in Fig. 3a. The estimates within these size ranges were more homogeneous (I^2^ =0.0%, p ranging from 0.524 to 0.883) than the overall estimates; hence, a fixed-effects model was applied. Growth rate increased with increased aneurysm diameter. The pooled mean growth rates for individuals with baseline diameters measuring 3.0–3.9 cm, 4.0–5.9 cm and ≥ 6.0 cm were 0.21 cm/year (95% CI: 0.19 cm/year to 0.23 cm/year), 0.38 cm/year (95% CI: 0.33 cm/year to 0.43 cm/year), and 0.71 cm/year (95% CI: 0.64 cm/year to 0.77 cm/year), respectively. AAA diameters at baseline were divided into 2.1–2.9 cm, 3.0–4.9 cm, and ≥ 5.0 cm in a study by Li et al. [35] and into 3.0–4.9 cm and ≥ 5.0 cm by Wu et al. [36]. Estimates of mean AAA growth rate at common baselines, namely 3.0–4.9 cm and ≥ 5.0 cm, are summarized in Fig. 3b. Random-effects meta-analysis was performed owing to substantial heterogeneity (I^2^ ranging from 97.4 to 98.2%). The pooled mean growth rates for individuals with baseline AAA diameters measuring 3.0–4.9 cm and ≥ 5.0 cm were 0.28 cm/year (95% CI: − 0.06 cm/year to 0.61 cm/year) and 0.75 cm/year (95% CI: 0.20 cm/year to 1.3 cm/year), respectively.

**Fig. 4:**
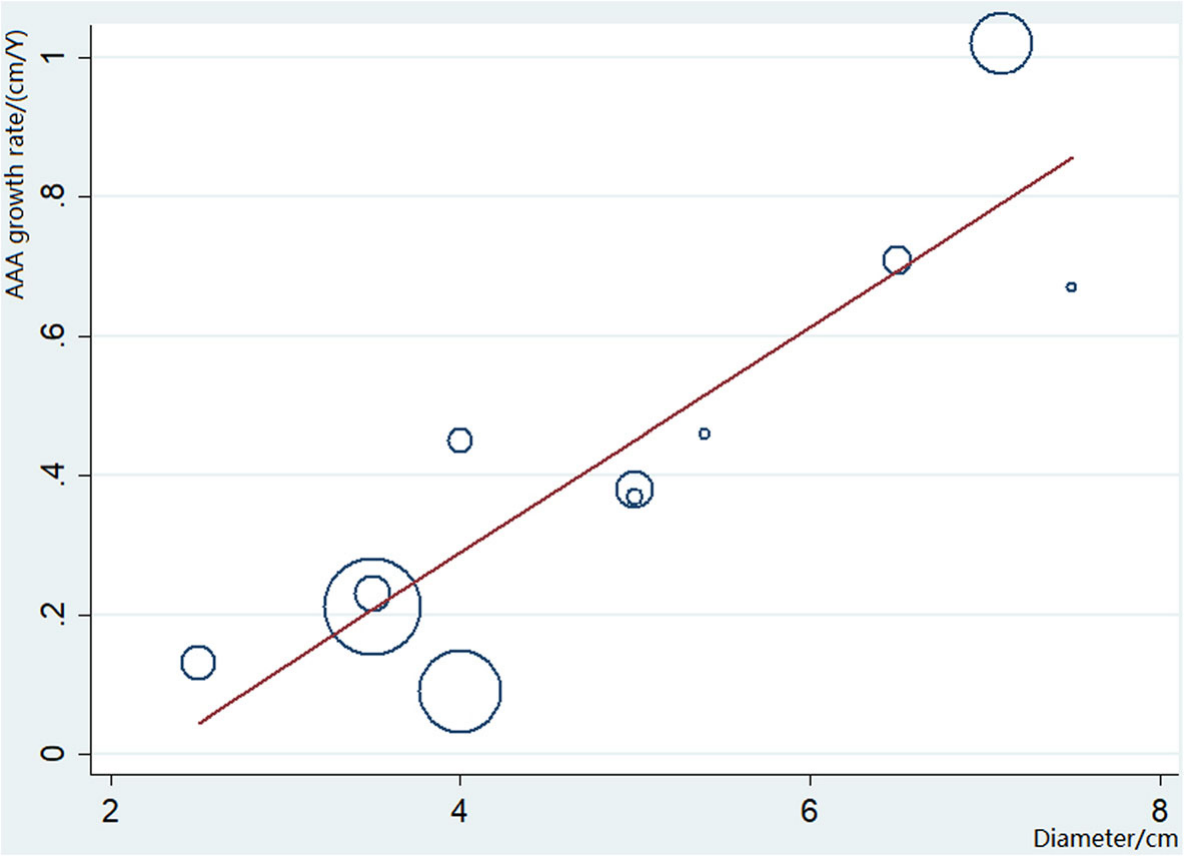
Meta-regression of AAA growth rate estimates by aneurysm diameter. The mid-point of the reported size range was used to calculate AAA diameter for range-specific estimates. Whereas the mean baseline diameter is used for overall estimates. The overall regression line is shown by the solid bold and the circles have an area in proportion to the amount of information

Meta-regression showed no statistically significant linear relationship between AAA growth rate and the covariates of age and female proportion, indicating that these might not be the sources of heterogeneity. The mid-point of the reported size range and the corresponding growth rate were used to perform meta-regression (Fig. 4), which showed a strong trend of linear relationship between AAA growth rate and aneurysm diameter. A total 77.48% of the variance between size ranges can be explained by the difference in aneurysm diameter. Based on the linear relationship between growth rate and aneurysm diameter, the size of AAA can be expected to grow exponentially with time. Given the meta-regression relationship shown, aneurysms with diameters of 3.0, 3.5, 4.0, 4.5, and 5.0 cm would be expected to take an average of 9.68, 5.80, 3.77, 2.24, and 1.02 years, respectively, to reach 5.5 cm.

## Discussion

This is the first systemic review of AAA growth rate in the Chinese population. In the review, the eligibility criteria for studies had to be somewhat flexible because relevant studies were very limited. We found the growth rates of AAA in Chinses population is ranging from 0.18 cm/year to 0.75 cm/year and that the growth rate increased with increased aneurysm diameter. Aneurysm diameter was one of the major sources of heterogeneity between studies.

Methods of growth rate estimation have been reported to influence aneurysm growth rate [38]; however, estimation methods were not reported in any of the four included studies. Exclusion of the only prospective study while performing sensitivity analysis did not substantially reduce the heterogeneity, indicating that study type may not be the source of heterogeneity. Sex is another factor that was reported to influence aneurysm growth rates, yet, because only one of the four included studies reported aneurysm growth rate in female patients and male patients separately, subgroup meta-analysis by pooling aneurysm growth rate in different sex could not be conducted. Cardiovascular risk factors, like smoking, and co-morbidities, such as hypertension and diabetes mellitus, were reported to influence growth rates of AAA. The influence of these factors on AAA growth rates were not evaluated in all four studies and so further estimation and analysis could not be conducted.

Thompson et al. reported that the overall AAA growth rate among Europeans and Americans was 0.232 cm/year [31], whereas our estimate of overall AAA growth rate was 0.43 cm/year. In Thompson’s study, most of the included studies were restricted to small abdominal aortic aneurysm (sAAA) and aneurysm diameter whereas none of the studies included in our meta-analysis were restricted to sAAA. Because aneurysm diameter is one of the main factors that influence aneurysm growth rate, this difference between Thompson’s study and ours may partially be caused by baseline diameters. To investigate whether race influences aneurysm growth rate, we compared the aneurysm growth rates in Chinese population and in western population after matching aneurysm diameters (3.0–3.9 cm or 3.0–4.9 cm). For aneurysm measuring 3.0–3.9 cm, the growth rates in western population and in Chinese population are 0.15 cm/year (95% CI: 0.09 to 0.20 cm/year, I^2^ =96.3%) and 0.21 cm/year (95% CI: 0.19 to 0.23 cm/year, I^2^ =0.0%), respectively. For aneurysm measuring 3.0–4.9 cm, the growth rates in western population and in Chinese population are 0.21 cm/year (95% CI: 0.12 to 0.30 cm/year, I^2^ =97.9%) and 0.28 cm/year (95% CI: − 0.06 to 0.61 cm/year, I^2^ =98.2%), respectively. Nevertheless, in order to match aneurysm diameters, only two studies can be used to estimate pooled aneurysm growth rates in Chinese patients, and one of the results has a wide range of 95% CI (− 0.06 to 0.61 cm/year) and hence is not statistically significant. Besides, in Tompson’s study, about half of patients are smokers, whereas in our study, in two of the four studies included, there are higher proportions of smokers while in the others two studies, patients’ smoking status was not described. Also, most patients in our study are with hypertension whereas only about one third patients in Tompson’s study are with hypertension. The difference of these risk factors in two population may also contribute to different growth rates. However, the information available is not sufficient for us to compare the AAA growth rates after matching risk factors. So this results do not necessarily mean that aneurysm growth rate is higher in Chinese patients than in western patients. More studies are required to elucidate the influence of race on aneurysm growth rate.

From the results of meta-regression, sex and age were not sources of heterogeneity. Normally, at least 10 studies are required to perform meta-regression. Therefore, the result of meta-regression of sex and age on AAA growth rate was unreliable, which means sex and age do not necessarily exert no influence on AAA growth rate. Although the meta-regression showed a strong trend of linear relationship between AAA growth rate and aneurysm diameter, none of the four included studies was specific to sAAA, and the sizes in these studies were less than 100. Hence, the findings of the current study are insufficient to put forward appropriate surveillance intervals for sAAA.

There is much work that remains to be done to help improve understanding of the epidemiological features of AAA in the Chinese population. There is no large-scale population screening of AAA at present in China; therefore, the prevalence rate of AAA in Chinese people is unclear. Multicenter studies investigating the growth and rupture rates of AAA, especially sAAA, are needed, to provide a basis for establishing a surveillance scheme and choosing suitable intervention times for Chinese patients. Currently, most of the related studies are retrospective. More prospective studies are needed that include standardized imaging processes, imaging performed independently by different people, and controlled follow-up frequency, to improve the quality of studies.

## Conclusion

In summary, we found that the growth rates of AAA in the Chinese population increase with AAA enlargement and appear to range from 0.18 cm/year in the smallest AAAs to 0.75 cm/year when the diameter exceeds 6 cm. However, based on current studies, it is difficult to estimate the accurate average AAA growth rate in Chinese patients. More large-scale, high-quality studies are required to achieve that. Overall, AAA growth rate increase with increased aneurysm diameter.

## Additional file

Additional file 1: **Table S1.** Excluded full-text articles with reasons for exclusion. (DOCX 13 kb)

## Data Availability

All data and materials are available.

## Abbreviations

AAA: Abdominal aortic aneurysm
CI: Confidence interval
CT: Computed tomography
Obs: Observational study
P: Prospective study
R: Retrospective study
RCT: Randomized controlled trial
sAAA: Small abdominal aortic aneurysm
SD: Standard deviation
US: Ultrasound

## References

[1] Nordon IM, Hinchliffe RJ, Loftus IM, Thompson MM (2011). Pathophysiology and epidemiology of abdominal aortic aneurysms. Nat Rev Cardiol.

[2] Golledge J, Kuivaniemi H (2013). Genetics of abdominal aortic aneurysm. Curr Opin Cardiol.

[3] Hellenthal FA, Buurman WA, Wodzig WK, Schurink GW (2009). Biomarkers of AAA progression. Part 1: extracellular matrix degeneration. Nat Rev Cardiol.

[4] Hellenthal FA, Buurman WA, Wodzig WK, Schurink GW (2009). Biomarkers of abdominal aortic aneurysm progression. Part 2: inflammation. Nat Rev Cardiol.

[5] Wassef M, Baxter BT, Chisholm RL, Dalman RL, Fillinger MF, Heinecke J (2001). Pathogenesis of abdominal aortic aneurysms: a multidisciplinary research program supported by the National Heart, Lung, and Blood Institute. J Vasc Surg.

[6] Wanhainen A, Verzini F, Van Herzeele I, Allaire E, Bown M, Cohnert T (2019). Editor's choice - European Society for Vascular Surgery (ESVS) 2019 clinical practice guidelines on the Management of Abdominal Aorto-iliac Artery Aneurysms. Eur J Vasc Endovasc Surg.

[7] Wilmink AB, Quick CR (1998). Epidemiology and potential for prevention of abdominal aortic aneurysm. Br J Surg.

[8] Stuntz M (2016). Modeling the burden of abdominal aortic aneurysm in the USA in 2013. Cardiology..

[9] Guirguis-Blake JM, Beil TL, Senger CA, Whitlock EP (2014). Ultrasonography screening for abdominal aortic aneurysms: a systematic evidence review for the U.S. preventive services task force. Ann Intern Med.

[10] Roger VL, Go AS, Lloyd-Jones DM, Benjamin EJ, Berry JD, Borden WB (2012). Heart disease and stroke statistics--2012 update: a report from the American Heart Association. Circulation..

[11] Bjorck M (2014). Surgery for ruptured abdominal aortic aneurysm. BMJ..

[12] United Kingdom Small Aneurysm Trial P (2002). Long-term outcomes of immediate repair compared with surveillance of small abdominal aortic aneurysms. N Engl J Med.

[13] Duan Q, Mao X, Liao C, Zhou H, Sun Z, Deng X (2016). Inhibition of BET bromodomain attenuates angiotensin II induced abdominal aortic aneurysm in ApoE(−/−) mice. Int J Cardiol.

[14] Yu J, Liu R, Huang J, Wang L, Wang W (2017). Inhibition of phosphatidylinositol 3-kinease suppresses formation and progression of experimental abdominal aortic aneurysms. Sci Rep.

[15] Yu J, Liu S, Huang J, Wang W (2018). Current theories and clinical trial evidence for limiting human abdominal aortic aneurysm growth. Curr Drug Targets.

[16] Huang J, Li G, Wang W, Wu K, Le T (2017). 3D printing guiding stent graft fenestration: a novel technique for fenestration in endovascular aneurysm repair. Vascular..

[17] Wang W, Xu B, Xuan H, Ge Y, Wang Y, Wang L (2018). Hypoxia-inducible factor 1 in clinical and experimental aortic aneurysm disease. J Vasc Surg.

[18] Greenhalgh RM, Powell JT (2008). Endovascular repair of abdominal aortic aneurysm. N Engl J Med.

[19] Chaikof EL, Dalman RL, Eskandari MK, Jackson BM, Lee WA, Mansour MA (2018). The Society for Vascular Surgery practice guidelines on the care of patients with an abdominal aortic aneurysm. J Vasc Surg.

[20] The UK Small Aneurysm Trial Participants (1998). Mortality results for randomised controlled trial of early elective surgery or ultrasonographic surveillance for small abdominal aortic aneurysms. Lancet..

[21] Hirsch AT, Haskal ZJ, Hertzer NR, Bakal CW, Creager MA, Halperin JL (2006). ACC/AHA 2005 guidelines for the management of patients with peripheral arterial disease (lower extremity, renal, mesenteric, and abdominal aortic): executive summary a collaborative report from the American Association for Vascular Surgery/Society for Vascular Surgery, Society for Cardiovascular Angiography and Interventions, Society for Vascular Medicine and Biology, Society of Interventional Radiology, and the ACC/AHA Task Force on Practice Guidelines (Writing Committee to Develop Guidelines for the Management of Patients With Peripheral Arterial Disease) endorsed by the American Association of Cardiovascular and Pulmonary Rehabilitation; National Heart, Lung, and Blood Institute; Society for Vascular Nursing; TransAtlantic Inter-Society Consensus; and Vascular Disease Foundation. J Am Coll Cardiol.

[22] Sakalihasan N, Limet R, Defawe OD (2005). Abdominal aortic aneurysm. Lancet..

[23] LaMorte WW, Scott TE, Menzoian JO (1995). Racial differences in the incidence of femoral bypass and abdominal aortic aneurysmectomy in Massachusetts: relationship to cardiovascular risk factors. J Vasc Surg.

[24] Johnson GJ, Avery A, McDougal EG, Burnham SJ, Keagy BA (1985). Aneurysms of the abdominal aorta. Incidence in blacks and whites in North Carolina. Arch Surg.

[25] Collins TC, Johnson M, Daley J, Henderson WG, Khuri SF, Gordon HS (2001). Preoperative risk factors for 30-day mortality after elective surgery for vascular disease in Department of Veterans Affairs hospitals: is race important?. J Vasc Surg.

[26] Arnaoutakis DJ, Propper BW, Black JR, Schneider EB, Lum YW, Freischlag JA (2013). Racial and ethnic disparities in the treatment of unruptured thoracoabdominal aortic aneurysms in the United States. J Surg Res.

[27] Banzic I, Lu Q, Zhang L, Stepak H, Davidovic L, Oszkinis G (2016). Morphological differences in the Aorto-iliac segment in AAA patients of Caucasian and Asian origin. Eur J Vasc Endovasc Surg.

[28] Guo W (2008). Guidelines for the diagnosis and treatment of abdominal aortic aneurysm. Chin J Practical Surg.

[29] Chiang CE, Wang TD, Li YH, Lin TH, Chien KL, Yeh HI (2010). 2010 guidelines of the Taiwan Society of Cardiology for the management of hypertension. J Formos Med Assoc.

[30] Moher D, Liberati A, Tetzlaff J, Altman DG (2010). Preferred reporting items for systematic reviews and meta-analyses: the PRISMA statement. Int J Surg.

[31] Thompson SG, Brown LC, Sweeting MJ, Bown MJ, Kim LG, Glover MJ (2013). Systematic review and meta-analysis of the growth and rupture rates of small abdominal aortic aneurysms: implications for surveillance intervals and their cost-effectiveness. Health Technol Assess.

[32] Fan LH, Chen FZ, Ye JR (1999). Natural history of abdominal aortic aneurysm. Chin J Surg.

[33] Zhao B, Zhou YX, Jiang YH (2008). Ultrasonography surveillance for senile and presenile asymptomatic abdominal arotic aneurysm. Med J Natl Defending Forces Northwest China.

[34] Zhang LF, Yao KC, Wang XH, Guo L, Wu D (2006). Long-term ultrasonography surveillance for non-surgical senile abdominal aortic aneurysm. Med J Chin People's Liberation Army.

[35] Li J, Li B, Liu DP (2008). Clinical observation for senile abdominal aortic aneurysm. Chin J Geriatr.

[36] Wu Q, Guo YT, Zhao YX, Zhang L (2009). Clinical analysis of 66 seline abdominal aortic aneurysm cases. Chin J Cardiovasc Med.

[37] Song HG (2013). Clinical analysis of ultrasonography surveillance for non-surgical senile abdominal aortic aneurysm. Chin Foreign Med Res.

[38] Brady AR, Thompson SG, Fowkes FG, Greenhalgh RM, Powell JT (2004). Abdominal aortic aneurysm expansion: risk factors and time intervals for surveillance. Circulation..

