# Supplementary material for: Meta-analysis of the growth rates of abdominal aortic aneurysm in the Chinese population
Source: BMC Cardiovasc Disord. 2019 Aug 22;19:204. doi: 10.1186/s12872-019-1160-x (PMC6704678; doi:10.1186/s12872-019-1160-x)
Supplement: Supplementary file 1 — Table S1. Excluded full-text articles with reasons for exclusion. (DOCX 13 kb) [file 12872_2019_1160_MOESM1_ESM.docx]

| Study | Title | First author | Journal/Year | Reason for exclusion |
| --- | --- | --- | --- | --- |
| (1) | Long-term ultrasonography surveillance for non-surgical senile abdominal aortic aneurysm | Zhang, F. L. | 2006 International Seminar on ultrasonography of vessels and superficial organs in Beijing /2006 | Data duplicated in another study |
| (2) | A ten-year ultrasound follow-up for 66 seline abdominal aortic aneurysm cases | Peng, y. | Chinese Journal of Geriatrics /2005 | Patients duplicated in another study |
| (3) | Abdominal aortic aneurysm in Chinese patients | Wei, W. I. | British Journal of Surgery/1985 | Not longitudinal study |
| (4) | Computed wall stress may predict the growth of abdominal aortic aneurysm | Li, Z. Y. | Annual International Conference of the IEEE Engineering in Medicine & Biology Society/ 2010 | Not restricted to Chinese population |
| (5) | Comparison of Progression Rate Between Thoracic And Abdominal Aortic Diameter in a High-risk Asian Population for Aortic Aneurysm: a Prospective, 3 Year Follow-up Study With Non-contrast Computed Tomography | Cho, I. | Circulation/2016 | Not restricted to Chinese population |
| (6) | Association between aneurysm shoulder stress and abdominal aortic aneurysm expansion: a longitudinal follow-up study | Li, Z. Y. | Circulation/2010 | Not restricted to Chinese population；Data duplicated in another study |
| (7) | Clinico-epidemiological features of infrarenal abdominal aortic aneurysm and relevant prognostic factors | Song, J. Q. | Zhonghua Yi Xue Za Zhi/2008 | Not longitudinal study |
| (8) | Association between use of oral-antidiabetic drugs and the risk of aortic aneurysm: a nested case-control analysis | Hsu, C. Y. | Cardiovascular Diabetology/2016 | Not longitudinal study |
| (9) | Long term outcomes in men screened for abdominal aortic aneurysm: prospective cohort study | Duncan J. L. | British Medical Journal/2012 | Not restricted to Chinese population |
| (10) | Cohort study of abdominal aortic aneurysm in women |  | Chinese Medical Information Report /2008 | Not restricted to Chinese population |
| (11) | Growth rate of infrarenal abdominal aortic aneurysm | Li, C. | Chinese Journal of Practical Surgery /1997 | Not restricted to Chinese population |
| (12) | Imaging surveillance of natural history of abdominal aortic aneurysm | Xu, Y. | International Journal of Surgery /1994 | Not restricted to Chinese population |
| (13) | Evaluation of abdominal aortic aneurysm growth rate with CT | Li, Q.S. | Diagnostic Imaging & Interventional Radiology /1993 | Not restricted to Chinese population |
| (14) | Diagnosis and treatment for abdominal aortic aneurysm | Lai, C.S. | Journal of Abdominal Surgery /1992 | Too few patients (n=5) |

**Additional file 1: Table S1.** Excluded full-text articles with reasons for exclusion.
